# Metagenomic analysis of fecal samples in colorectal cancer Egyptians patients post colectomy: A pilot study

**DOI:** 10.3934/microbiol.2024008

**Published:** 2024-02-20

**Authors:** Rana H. Abo-Hammam, Mohammed Salah, Sarah Shabayek, Amro Hanora, Samira Zakeer, Randa H. Khattab

**Affiliations:** 1 Forensic toxicologist and narcotics expert, Ministry of Justice, Tanta, Egypt; 2 Department of Microbiology and Immunology, Faculty of pharmacy, Port-Said University, Port-Said, Egypt; 3 Department of Microbiology and Immunology, Faculty of pharmacy, Suez Canal University, Ismailia, Egypt; 4 Department of Microbiology and Immunology, Al-Salam University, Tanta, Egypt

**Keywords:** CRC, gut microbiota, metagenomic, dysbiosis, tumor biomarker

## Abstract

One of the most prevalent malignancies that significantly affects world health is colorectal cancer (CRC). While genetics are involved in a portion of CRC patients, most cases are sporadic. The microbiome composition could be a new source of tumor initiation and progression. This research was conducted to investigate the microbiota composition of CRC patients post colectomy at taxonomic and functional levels. Using a next-generation sequencing approach, using an Illumina Novaseq 6000, the fecal samples of 13 patients were analyzed and the obtained data was subjected to a bioinformatics analysis. The bacterial abundance and uniqueness varied in CRC patients alongside differences in bacterial counts between patients. *Bacteroides fragilis*, *Bacteroides vulgatus*, *Escherichia coli*, and *Fusobacterium nucleatum* were among the pro-cancerous microorganisms found. Concurrently, bacteria linked to CRC progression were detected that have been previously linked to metastasis and recurrence. At the same time, probiotic bacteria such as *Bifidobacterium dentium*, *Bifidobacterium bifidum*, and *Akkermansia muciniphila* increased in abundance after colectomies. Additionally, numerous pathways were deferentially enriched in CRC, which emerged from functional pathways based on bacterial shotgun data. CRC-specific microbiome signatures include an altered bacterial composition. Our research showed that microbial biomarkers could be more usefully employed to explore the link between gut microbiota and CRC using metagenomic techniques in the diagnosis, prognosis, and remission of CRC, thereby opening new avenues for CRC treatment.

## 1. Introduction

Colorectal cancer (CRC) is one of the most common types of cancer worldwide; it is third in the world in terms of recognition (about 6.2%) and second in terms of mortality (about 10% of cancer-related mortality) [1]. It is the sixth most common cancer in both men and women in Egypt, accounting for 6.5% of all malignant tumors. These findings differ depending on the level of economic development in each country [2].

The human gut microbiota has a vital function in maintaining overall health and has been related to the emergence of conditions such as type 2 diabetes, colorectal cancer, and obesity [3]. Several findings focused on the complication and two-way correlation between microbiota and cancer. Cancer progression may alter the microbiota. Meanwhile, changing the microbiota may have an impact on cancer development [4]. The metabolites produced by the symbiotic bacteria that inhabit the human gut have long had an impact on the host's physiological and pathological processes, both locally and systemically. Various types of cancer may be significantly influenced by the microbiota in terms of their occurrence, progression, therapy, and prognosis. The development of the era of personalized therapies depend on an understanding of the functional role of the gut microbiota in cancer [5].

A microbiota imbalance favoring opportunistic infections causes increased mucosal permeability, bacterial translocation, and activation of both the innate and adaptive immune systems, which results in chronic inflammation. In contrast, inflammation cannot trigger CRC without the presence of either microbiota or microbial substances [6]. Chemotherapeutics have been utilized for many years and are still often employed as first-line therapies for a variety of human malignancies, including CRC. Yet, a sizable portion of patients using these medications are likely to experience treatment-related morbidities and mortality. Currently, studies are focused on how the intestinal microbiota affects the effectiveness and toxicity of the existing chemotherapeutic medicines because CRC occurs near gut bacteria [7].

Using next-generation sequencing technology to investigate the microbiomes of various species and environments has gained a greater understanding of the metabolic, physiological, and ecological roles of environmental microorganisms. Nowadays, Illumina systems are widely used to analyze metagenomics samples from a variety of environments [8].

Regretfully, in Egypt, the majority of CRC cases undergo advanced-stage diagnoses. Although a colonoscopy is a gold standard for diagnosis, it has several drawbacks, including being an expensive and painful diagnostic procedure. Therefore, the necessity for effective non-invasive diagnostic methods is paramount. According to studies, the composition and diversity of the gut microbiota varies between healthy individuals and CRC patients, and these variations may serve as biomarkers for early, noninvasive CRC detection, which can lower the incidence and mortality of CRC. These discoveries have garnered interest due to their potential for sensitivity, specificity, and even cost-effectiveness [9].

Consequently, this study examined the taxonomic and functional changes in the bacterial composition of the intestine in CRC patients in an effort to transform the way CRC is diagnosed and to show how treatment affects the variety of intestinal bacteria present in patients with CRC.

## 2. Materials and methods

### 2.1. Patients and samples collection

The research ethics committee (Reference code: 2020MH1) of the faculty of pharmacy at Suez Canal University in Egypt provided recommendations for the study's design. Thirteen patients, aged 55 to 60, who had been diagnosed with cancer by Tanta City's oncology center were included in the study after providing their informed consent. Fresh fecal samples from the participants were promptly sub-packed and labeled. All tubes were maintained at −80 C. The clinical and pathological characteristics of CRC patients, as well as their course of treatment, are displayed in Table 1.

**Table 1.** The clinical-pathological characteristics of CRC patients and their treatment lines.

Study group	Patients IDs	Sex	Treatment
Category 1	59483	Male	Colectomy (was done less than one year ago) + adjuvant folfox^1^
	59489	Female	
	59492	Female	
	59494	Male	
	59496	Female	
	59497	Female	
Category 2	59498	Male	Follow up (colectomy was done more than one year ago) +adjuvant folfiri^2^
	59499	Male	
	59500	Male	
	59482	Male	
	59484*	Female	
	59486*	Female	
	59485**	Male	

1 folfox is a chemotherapy regimen consisting of folinic acid (leucovorin), fluorouracil (5fu) and oxaliplatin.

2 folfiri is a chemotherapy regimen consisting of leucovorin calcium (calcium folinate), 5-fluorouracil, and irinotecan.

*these patients developed a recurrent CRC.

**this patient developed a liver metastatic CRC and take xelox a combined therapy of oxaliplatin and capectabine beside folfiri regimen.

### 2.2. Metagenomics analysis

#### 2.2.1. DNA extraction, purification, and bacterial whole genome sequencing

Total fecal DNA extraction was performed using the QIAamp PowerFecal DNA Kit from QIAGEN in Germany (cat. no. 12830-50). All extracted DNA samples were evaluated using a Qubit dsDNA kit and a Nanodrop to ensure that there was an adequate amount and quality of input DNA for the shotgun sequencing. The DNA sequencing was conducted by IGA technology services (Audin, Italy); then, the library was sequenced on a Novaseq 6000 in paired end 150 bp mode.

### 2.3. Bioinformatics analysis

Raw sequence files were used to begin the bioinformatics analysis. FASTQC was used to check the quality of the reads before filtering out the low-quality reads and adaptor sequences. For paired end reads, Trimmomatic v0.39 [10] was used. Kneaddata v0.33 was used to remove the host reads. MetaPhlAn 3.0 was used for a metagenomic phylogenetic analysis [11], and a table containing the detected microbes and their relative abundances was produced. GraPhlAn 2.7 [12] generated circular representations of taxonomic and phylogenetic trees for phylogenetic visualization. The metabolic analysis network was detected using HUMAnN2 3.0 for functional profiling [13]. Its pipeline began with a quality-controlled DNA sequence, followed by MetaPhlAn3 to detect microbes and generate a list of microbial abundance, and bowtie2 was used to detect the nucleated-level pangenome. The algorithm was divided into two branches: one for mapped reads and one for unmapped reads. The diamond algorithm detected hit protein families using unmapped reads. The core HUMAnN algorithm was applied to both files to generate the gene family abundance, pathway abundance, and pathway coverage. All tools were constructed using the default settings.

### 2.4. Species diversity analysis

#### 2.4.1. Alpha diversity

An alpha diversity analysis was performed using R packages such as phyloseq, ranacapa, and ggplot (CRAN package). Moreover, alpha indices such as Observed, Simpson, and Fisher were used. In addition, the rarefaction curve was plotted to represent the number of species (species richness) as a function of the number of samples (sequence sample size).

#### 2.4.2. Beta diversity

The beta diversity was measured in different distances metrics: Quantitative metrics (e.g., weighted UniFrac) determined the differences in the abundance of the individuals between samples/groups; and Qualitative metrics (e.g., unweighted UniFrac) determined either the presence or absence of the individuals between samples/groups. The un/weighted UniFrac is illustrated by a heatmap, where the numbers in the grid show the difference of coefficient between two samples/groups. A beta diversity analysis was performed using a PCoA (principal coordinate analysis) to show the dissimilarity (presence/absence) of the amplicon sequence variant (ASVs) between samples.

### 2.5. Functional analysis

For functional analysis visualization, the Ggplot and circlize packages in R were used to generate a chord diagram and a pathways heatmap, respectively.

### 2.6. Correlation analysis

The method used df. corr (method = "pearson") in Python (https://pandas.pydata.org/pandas-docs/stable/reference/api/pandas.DataFrame.corr.html).

## 3. Results

### 3.1. Taxonomic results

Using metagenome sequencing data to investigate the bacterial fraction of the microbiota, at the phylum level, there were 10 intestinal bacteria phyla among the CRC patients (Ascomycota, Eukaryota unclassified, Euryarchaeota, Bacteroidetes, Firmicutes, Proteobacteria, Actinobacteria, Verrucomicrobia, Fusobacteria, and Lentisphaerae). Both male patients (59494 & 59482) from the two-study groups had Proteobacteria as a highly abundant phylum. Firmicutes were highly abundant in only two patients (59484 & 59483); in the remaining patients, Bacteriodetes was the abundant phylum, as shown in was the abundant phylum.

**Figure 1.**
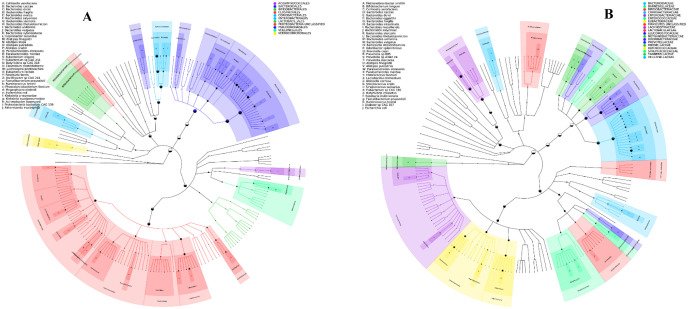
A) Phylogenetic tree represent the genus and species in category 1 CRC patients. B) Phylogenetic tree represent the genus and species in category 2 CRC patients.

A total of 101 genera and 262 species were present in all CRC patients. For patients in both groups 1 & 2, *E. coli* was the most abundant species. However, *Bacteriode vulgatus* was detected with a varying abundance. In addition to these bacteria, *Bacteroides fragilis* and *Fusobacterium nucleatum* were detected with a high abundance in a recurrent CRC patient (I.D. 59486). The abundance of beneficial genera *Bifidobacterium* and *Akkermansia* were prevalent in colectomy patients. Moreover, the *Lactobacillus* genus was higher in relative abundance in follow-up patients. The most abundant genus and species for the two CRC groups were represented in the phylogenetic tree (Figure 1). The unique microbial species for each category are listed in Table S1.

**Figure 2.**
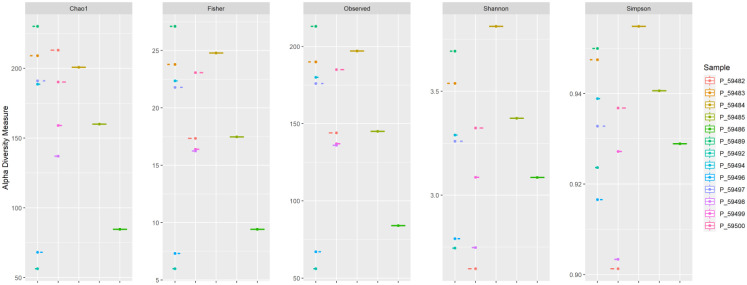
Alpha diversity indices.

The number of species observed in a patient diagnosed with a recurrent state within group 2 (i.d. 59486) was low, and two female patients (i.d. 59496 and 59492) within category 1 were the lowest. According to the alpha diversity indices, a patient diagnosed with a recurrent state within group 2 (i.d. 59486) was the lowest among all indices. According to the Shannon and Simpson indices, other patients also diagnosed with a recurrent CRC state (i.d. 59484) were the highest in diversity (Figure 2).

**Figure 3.**
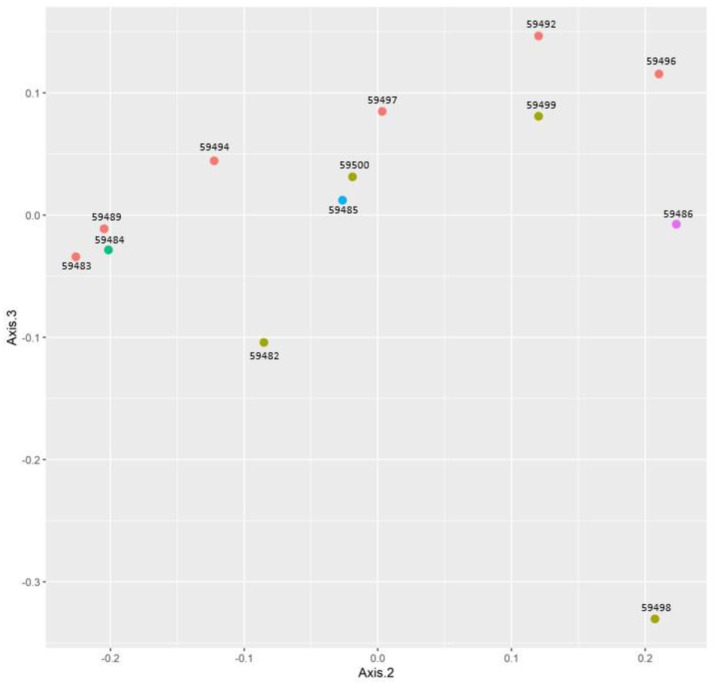
Principal Coordinate Analysis (PCoA).

The beta diversity indices measured based on weighted uniFrac and unweighted uniFrac distances were represented, where the smaller the number, the smaller the difference in the species diversity between the two samples/groups. The weighted unifrac distance between follow-up patients and colectomy patients was the lowest. Alternatively, the highest distance was between patients in a recurrent state and other follow-up patients. The unweighted uniFrac distance between patients diagnosed with a recurrent state within group 2 (i.d. 59486) and colectomy patients was the highest (Figure S1). Concerning the PcoA, it showed that samples from the same group were not clustered together (Figure 3).

### 3.2. Functional analysis

**Figure 4.**
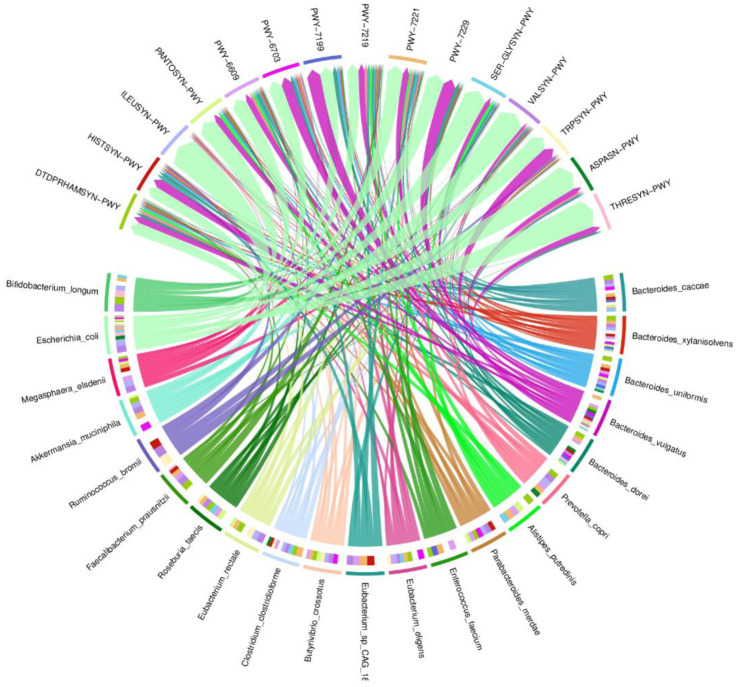
A circle diagram showing the abundance of bacterial species in the selected pathways.

A chord diagram was used to express the connections between bacterial species and their pathways (Figure 4). Twenty-one bacterial species were selected to show their abundances in 15 pathways as follows: ILEUSYN-PWY:L-isoleucine biosynthesis I (from threonine), VALSYN-PWY: L-valine biosynthesis, PWY-7199: pyrimidine deoxyribonucleosides salvage, PWY-6609: adenine and adenosine salvage III, PANTOSYN-PWY: superpathway of coenzyme A biosynthesis I (bacteria), HISTSYN-PWY: L-histidine biosynthesis, THRESYN-PWY: superpathway of L-threonine biosynthesis, SER-GLYSYN-PWY: superpathway of L-serine and glycine biosynthesis I, PWY-7219: adenosine ribonucleotides de novo biosynthesis, PWY-7221: guanosine ribonucleotides de novo biosynthesis, PWY-7229: superpathway of adenosine nucleotides de novo biosynthesis I, ASPASN-PWY: superpathway of L-aspartate and L-asparagine biosynthesis, TRPSYN-PWY: L-tryptophan biosynthesis, PWY-6703: preQ0 biosynthesis, and DTDPRHAMSYN-PWY: dTDP-&beta;-L-rhamnose biosynthesis. As shown in the figure, *E.coli* was the most abundant species, followed by *Bacteroides vulgatus* participating in most pathways.

The functional characteristics of the gut microbiome differed between CRC patients' metabolic pathways (Figure S2). The super pathway of purine nucleotides de novo biosynthesis II was not detected in either group 1 or group 2, except for patients in a recurrent state and patients diagnosed with liver metastatic CRC from group 2. In addition, these patients had low abundances of the super pathways of glycolysis, pyruvate dehydrogenase, TCA, and glyoxylate bypass; moreover, this pathway had a zero abundance in the remaining patients of the follow-up groups (59483&59489).

### 3.3. Correlation analysis

The taxonomy for OTU which correlated with samples is listed in Table S2. Both groups 1 and 2 had a negative correlation, −0.617, which can indicate that there was an increase in group 1 alongside a decrease in group 2. OTU255 (0.451) and OTU256 (0.447) had a positive correlation to group 1. Based on Table 2, both OTU are from the *Roseburia* genus. Additionally, OTU171 (0.569) and OTU175 (0.569) had a positive correlation to group 2; both OTU are from the *Streptococcaceae* family. Moreover, OTU350 (0.459) and OTU348 (0.437) had a positive correlation to this study group; both OTU are from the *Dialister* genus. Specifically, OTU229 (0.964) and OTU228 (0.920) had a positive correlation with patients who developed recurrent CRC (group 2); OTU335 (0.934) had a positive correlation with liver metastatic CRC patients. Concerning the sex effect, both OTU98 (0.570) and OTU75 (0.506) had a positive correlation with female patients, while OTU171 (0.569) and OTU175 (0.569) had a positive correlation with male patients.

## 4. Discussion

Through its involvement in immunological control, drug metabolism, and food absorption in the human body, the gut microbiota plays a critical role in preserving the integrity of the intestinal micro-ecosystem [14].

At the species level and concerning the PcoA, it showed that samples from the same group were not clustered together, which can assume that the microbiome in each group were not similar. Besides that, we cannot distinctively classify samples from the same group based on the abundance of the bacteria. The CRC patients were characterized by a higher abundance of enterotoxigenic *Bacteroides fragilis*, especially in patients who developed a recurrent CRC (I.D. 59486). This may be related to metalloprotease toxin secreted by enterotoxigenic Bacteroides fragilis (ETBF), thereby resulting in genotoxicity, epithelial damage, and colorectal neoplasia [15].

Interestingly, the present results showed that the *Fusobacterium nucleatum* was well noted in patients who developed a recurrent CRC (I.D. 59486), but still lower in abundance compared to *E.coli*. However, Kostic et al revealed that *Fusobacterium nucleatum* was found to be persistent in both healthy and CRC hosts [16], thus indicating that its detection alone is not a reliable enough biomarker for CRC.

*Bacteriode vulgatus* was found in all CRC patients with varying abundances, while *Bacteriode caccae* was found in liver metastatic CRC patient group 2. Yet, it is known that *Bacteroides vulgatus* and *Bacteroides caccae* both contribute to the breakdown of the gut's colonic wall, which may contribute to the development of CRC [17].

*Porphyromonas gingivalis*, a bacterium with a strong connection to periodontal infection and a subsequent cause of numerous types of gastrointestinal cancers, is one of the distinctive microorganisms in Category 1. The potential for *Porphyromonas gingivalis* to cause CRC and the mechanism underlying such a promotion is still unknown. *Porphyromonas gingivalis* was shown to be prevalent in human feces and tissue samples from patients with CRC in 2021, in contrast to tissue samples from patients with either colorectal adenoma or healthy controls [18].

*Bifidobacterium bifidum*, which has anti-colon cancer activity and is described as a probiotic, was a unique bacterium in this category. However, it was quite low in abundance. In addition, *Enterococcus faecalis* was a unique bacterium signature for category 1. The literature implies that *Enterococcus faecalis* may play a deleterious role in CRC, though its exact function is yet unknown. Others have included *E. faecalis* as a significant probiotic microbe [19].

*Akkermansia muciniphila* was unique in category 2 patients, though it was very low in abundance. In CRC patients who received FOLFOX treatment, the abundance of *Akkermansia muciniphila* significantly increased and showed a favorable correlation with the therapeutic outcome. Additionally, *A*. *muciniphila* colonization markedly improved FOLFOX's anti-cancer effectiveness [20]. Rather than receiving FOLFOX, this patient group received FOLFIRI. Therefore, we suggest the FOLFOX regimen as a possible colon cancer treatment plan for this group. There were several distinct Actinomyces species in Category 2. Actinomycosis, which is an infection caused by Actinomyces, has previously been linked to symptoms that resemble cancer, particularly lymphoma [21].

*Clostridium lavalense* was a unique bacterial signature for liver metastatic CRC; however, it was very low in abundance. Moreover, it was reported that an intestinal malignancy was among the risk factors of Clostridia [22]. The archaeal species *Methanobrevibacter smithii* was the unique signature for a patient with a recurrence CRC state. This species is linked to an increase in the risk factor for CRC [23].

The functional characteristics of the gut microbiome differed between CRC patients' metabolic pathways, even in the same diagnostic groups. This may be related to other diseases besides colorectal cancer. Furthermore, the abundance of purine pathways was higher than that of pyrimidine, which supports the use of purine antimetabolites as antitumor medicines to treat malignancies by preventing DNA synthesis and slowing cell development [24].

The current research confirmed the findings of Thomas et al and Ma et al, who reported that the starch degradation route, the metabolism of nucleotides, the metabolism of energy, and the intermediates of amino acids were all affected [25],[26]. These findings imply that CRC patients' gut microbes utilize host amino acids differently from healthy individuals.

## 5. Limitations

One limitation of our study is the lack of resources to select an adequate sample size to successfully address microbial dysbiosis in colon cancer patients. Furthermore, funding was lacking to perform shotgun microbiome sequencing data at multiple time points before and after surgery to assess whether these patterns of dysbiosis persisted and correlated with the disease outcome.

## 6. Conclusion

Rather than specific pathogenetic microorganisms, the collective microbial dysregulation in interactions between a large number of microorganisms causes CRC, which includes marked increases and decreases in specific species. Although there are patients who have had colectomy surgery, microbial dysbiosis is still persistent. Future studies should use larger sample sizes and collect shotgun microbiome sequencing data at multiple time points pre- and post-surgery to assess whether these dysbiosis patterns persist and correlate with disease outcomes.



## References

[1] Guyton KZ, Loomis D, Grosse Y (2015). Carcinogenicity of tetrachlorvinphos, parathion, malathion, diazinon, and glyphosate. Lancet Oncol.

[2] Suliman MA, Zamzam ML, Omar AT (2020). Clinicopathological profile of colorectal cancer patients in Suez Canal University Hospitals-Egypt. J Cancer Biol Res.

[3] Liu R, Hong J, Xu X (2017). Gut microbiome and serum metabolome alterations in obesity and after weight-loss intervention. Nat Med.

[4] Zitvogel L, Daillère R, Roberti MP (2017). Anticancer effects of the microbiome and its products. Nat Rev Microbiol.

[5] Sun J, Chen F, Wu G (2023). Potential effects of gut microbiota on host cancers: focus on immunity, DNA damage, cellular pathways, and anticancer therapy. ISME J.

[6] Arthur JC, Perez-Chanona E, Mühlbauer M (2012). Intestinal inflammation targets cancer-inducing activity of the microbiota. Science.

[7] Dekker E, Tanis PJ, Vleugels JLA (2019). Colorectal cancer. Lancet.

[8] Bharti R, Grimm DG (2021). Current challenges and best-practice protocols for microbiome analysis. Brief Bioinform.

[9] Olovo CV, Huang X, Zheng X (2021). Faecal microbial biomarkers in early diagnosis of colorectal cancer. J Cell Mol Med.

[10] Bolger AM, Lohse M, Usadel B (2014). Trimmomatic: a flexible trimmer for Illumina sequence data. Bioinformatics.

[11] Beghini F, McIver LJ, Blanco-Míguez A (2021). Integrating taxonomic, functional, and strain-level profiling of diverse microbial communities with bioBakery 3. Elife.

[12] Asnicar F, Weingart G, Tickle TL (2015). Compact graphical representation of phylogenetic data and metadata with GraPhlAn. PeerJ.

[13] Abubucker S, Segata N, Goll J (2012). Metabolic reconstruction for metagenomic data and its application to the human microbiome. PLoS Comput Biol.

[14] Wieczorska K, Stolarek M, Stec R (2020). The role of the gut microbiome in colorectal cancer: where are we? where are we going?. Clin Colorectal Cancer.

[15] Zakharzhevskaya NB, Tsvetkov VB, Vanyushkina AA (2017). Interaction of bacteroides fragilis toxin with outer membrane vesicles reveals new mechanism of its secretion and delivery. Front Cell Infect Microbiol.

[16] Kostic AD, Chun E, Robertson L (2013). Fusobacterium nucleatum potentiates intestinal tumorigenesis and modulates the tumor-immune microenvironment. Cell Host Microbe.

[17] Sánchez-Alcoholado L, Ordóñez R, Otero A (2020). Gut microbiota-mediated inflammation and gut permeability in patients with obesity and colorectal cancer. Int J Mol Sci.

[18] Wang X, Jia Y, Wen L (2022). Porphyromonas gingivalis promotes colorectal carcinoma by activating the hematopoietic NLRP3 inflammasome. Cancer Res.

[19] Huycke MM, Abrams V, Moore DR (2002). Enterococcus faecalis produces extracellular superoxide and hydrogen peroxide that damages colonic epithelial cell DNA. Carcinogenesis.

[20] Hou X, Zhang P, Du H (2021). Akkermansia muciniphila potentiates the antitumor efficacy of FOLFOX in colon cancer. Front Pharmacol.

[21] Roh YH, Park KJ, Byun KD (2019). Abdominal actinomycosis misconceived as intestinal lymphoma: Report of a case. Int J Surg Case Rep.

[22] Garceau R, Bourque C, Thibault L (2016). First report of clostridium lavalense isolated in human blood cultures. Can J Infect Dis Med Microbiol.

[23] Gupta A, Dhakan DB, Maji A (2019). Association of *Flavonifractor plautii*, a flavonoid-degrading bacterium, with the gut microbiome of colorectal cancer patients in India. MSystems.

[24] Wang W, Cui J, Ma H (2021). Targeting pyrimidine metabolism in the era of precision cancer medicine. Front Oncol.

[25] Thomas AM, Manghi P, Asnicar F (2019). Author Correction: Metagenomic analysis of colorectal cancer datasets identifies cross-cohort microbial diagnostic signatures and a link with choline degradation. Nat Med.

[26] Ma Y, Zhang Y, Xiang J (2021). Metagenome analysis of intestinal bacteria in healthy people, patients with inflammatory bowel disease and colorectal cancer. Front Cell Infect Microbiol.

