# Supplementary material for: Metagenomic analysis of fecal samples in colorectal cancer Egyptians patients post colectomy: A pilot study
Source: AIMS Microbiol. 2024 Feb 20;10(1):148–60. doi: 10.3934/microbiol.2024008 (PMC10955169; doi:10.3934/microbiol.2024008)
Supplement: Supplementary file 1 [file microbiol-10-01-008-s001.pdf]

AIMS Microbiology, 10(1): 148–160.
DOI: 10.3934/microbiol.2024008
Received: 30 September 2023
Revised: 17 December 2023
Accepted: 29 December 2023
Published: 20 February 2024

http://www.aimspress.com/journal/microbiology

Research article

Metagenomic analysis of fecal samples in colorectal cancer Egyptians patients post colectomy: A pilot study.

Rana H. Abo-Hammam[1,†], Mohammed Salah[2], Sarah Shabayek[3], Amro Hanora[3,*], Samira Zakeer[3] and Randa H. Khattab[4,†]

[1] Forensic toxicologist and narcotics expert, Ministry of Justice, Tanta, Egypt
[2] Department of Microbiology and Immunology, Faculty of pharmacy, Port-Said University, Port-Said, Egypt
[3] Department of Microbiology and Immunology, Faculty of pharmacy, Suez Canal University, Ismailia, Egypt
[4] Department of Microbiology and Immunology, Al-Salam University, Tanta, Egypt

* **Correspondence:** Email: a.hanora@pharm.suez.edu.eg.

† These Authors are equally contributed to this manuscript.

Supplementary

Table S1. The unique bacterial species in CRC patients' categories.

Category 1	Category 2
Acinetobacter towneri	Actinobaculum oral taxon 183
Actinomyces ICM 47	Actinomyces graevenitzii
Bifidobacterium adolescentis	Actinomyces odontolyticus
Bifidobacterium bifidum	Actinomyces oris
Bifidobacterium catenulatum	Actinomyces HMSC035G02
Bifidobacterium pseudolongum	Actinomycesoral taxon 181
Rothia aeria	Actinomycesoral taxon 448

Continued on next page

Category 1	Category 2
Rothia mucilaginosa	Bifidobacterium_minimum
Collinsella intestinalis	Bifidobacterium mongoliense
Adlercreutzia equolifaciens	Scardovia wiggsiae
Bacteroides clarus	Corynebacterium_amycolatum
Bacteroides faecis	Corynebacterium HMSC08A12
Bacteroides faecis CAG 32	Cutibacterium acnes
Bacteroides galacturonicus	Propionibacterium freudenreichii
Bacteroides CAG 927	Bacteroides nordii
Porphyromonas gingivalis	Coprobacter_fastidiosus
Alistipes inops	Prevotella_sp_885
Alistipes onderdonkii	Prevotella AM42 24
Parabacteroides goldsteinii	Prevotella stercorea
Parabacteroides johnsonii	Gemella_haemolysans
Phascolarctobacterium CAG 266	Lactobacillus_agilis
Enterococcus avium	Lactobacillus fermentum
Enterococcus faecalis	Lactobacillus helveticus
Enterococcus gallinarum	Lactobacillus_mucosae
Enterococcus hirae	Lactobacillus_paragasseri
Lactobacillus ruminis	Lactobacillus_pontis
Streptococcus thermophilus	Lactobacillus_rossiae
Clostridium bolteae CAG 59	Weissella confusa
Clostridium CAG 253	Lactococcus chungangensis
Clostridium ventriculi	Streptococcus milleri
Bacteroides pectinophilus	Streptococcus mitis
Lawsonibacter asaccharolyticus	Streptococcus pasteurianus
Eubacterium CAG 251	Streptococcus vestibularis
Eubacterium OM08 24	Clostridium bolteae CAG 59
Blautia CAG 257	Eisenbergiella massiliensis
Clostridium aldenense	Clostridium_citroniae
Clostridium asparagiforme	Clostridium_lavalense
Clostridium clostridioforme	Romboutsia ilealis
Roseburia CAG 182	Anaerotruncus colihominis
Roseburia CAG 303	Flavonifractor An10
Sellimonas intestinalis	Catenibacterium mitsuokai
Clostridioides difficile	Coprobacillus cateniformis
Paeniclostridium sordellii	Firmicutes bacterium CAG 110
Anaeromassilibacillus An250	Phascolarctobacterium succinatutens
Pseudoflavonifractor An184	Veillonella parvula
Ruminococcaceae bacterium D5	Methylobacterium_frigidaeris
Ruminococcus obeum CAG 39	Achromobacter xylosoxidans
Ruminococcus CAG 403	Desulfovibrio_fairfieldensis
Ruminococcus CAG 488	Haemophilus parainfluenzae
Holdemanella biformis	Proteobacteria bacterium_CAG 139
Firmicutes bacterium CAG 94	Akkermansia muciniphila
Mitsuokella jalaludinii	Candida albicans
Mitsuokella multacida	Candida dubliniensis
Dialister invisus	Candida tropicalis
Dialister succinatiphilus	Saccharomyces cerevisiae
Megasphaera hexanoica	Fusobacterium varium
Veillonella dispar	
Veillonella infantium	
Veillonella T11011 6	

Continued on next page

Category 1	Category 2
Tissierella P1	
Lautropia mirabilis	
Comamonas kerstersii	
Desulfovibrionaceae bacterium	
Desulfovibrio piger	
Morganella morganii	
Proteus mirabilis	
Acinetobacter baumannii	
Paraprevotella clara	

Table S2. Taxonomy for OTU correlated with samples.

NCBI_ ID	Phylum	Class	Order	Family	Genus	Species
OTU48	Actinobacteria	Actinobacteria	Propionibacteriales			
OTU75	Bacteroidetes	Bacteroidia	Bacteroidales			
OTU80	Bacteroidetes	Bacteroidia	Bacteroidales	Bacteroidaceae	Bacteroides	Bacteroides dorei
OTU98	Bacteroidetes	Bacteroidia	Bacteroidales	Bacteroidaceae	Bacteroides	Bacteroides vulgatus
OTU135	Bacteroidetes	Bacteroidia	Bacteroidales	Tannerellaceae		
OTU136	Bacteroidetes	Bacteroidia	Bacteroidales	Tannerellaceae	Parabacteroides	
OTU171	Firmicutes	Bacilli	Lactobacillales	Streptococcaceae		
OTU175	Firmicutes	Bacilli	Lactobacillales	Streptococcaceae	Streptococcus	
OTU219	Firmicutes	Clostridia	Clostridiales	Lachnospiraceae		
OTU228	Firmicutes	Clostridia	Clostridiales	Lachnospiraceae	Butyrivibrio	
OTU229	Firmicutes	Clostridia	Clostridiales	Lachnospiraceae	Butyrivibrio	
OTU255	Firmicutes	Clostridia	Clostridiales	Lachnospiraceae	Roseburia	
OTU256	Firmicutes	Clostridia	Clostridiales	Lachnospiraceae	Roseburia	Roseburia faecis
OTU335	Firmicutes	Negativicutes	Acidaminococcales			
OTU348	Firmicutes	Negativicutes	Veillonellales	Veillonellaceae	Dialister	
OTU350	Firmicutes	Negativicutes	Veillonellales	Veillonellaceae	Dialister	Dialister CAG 357

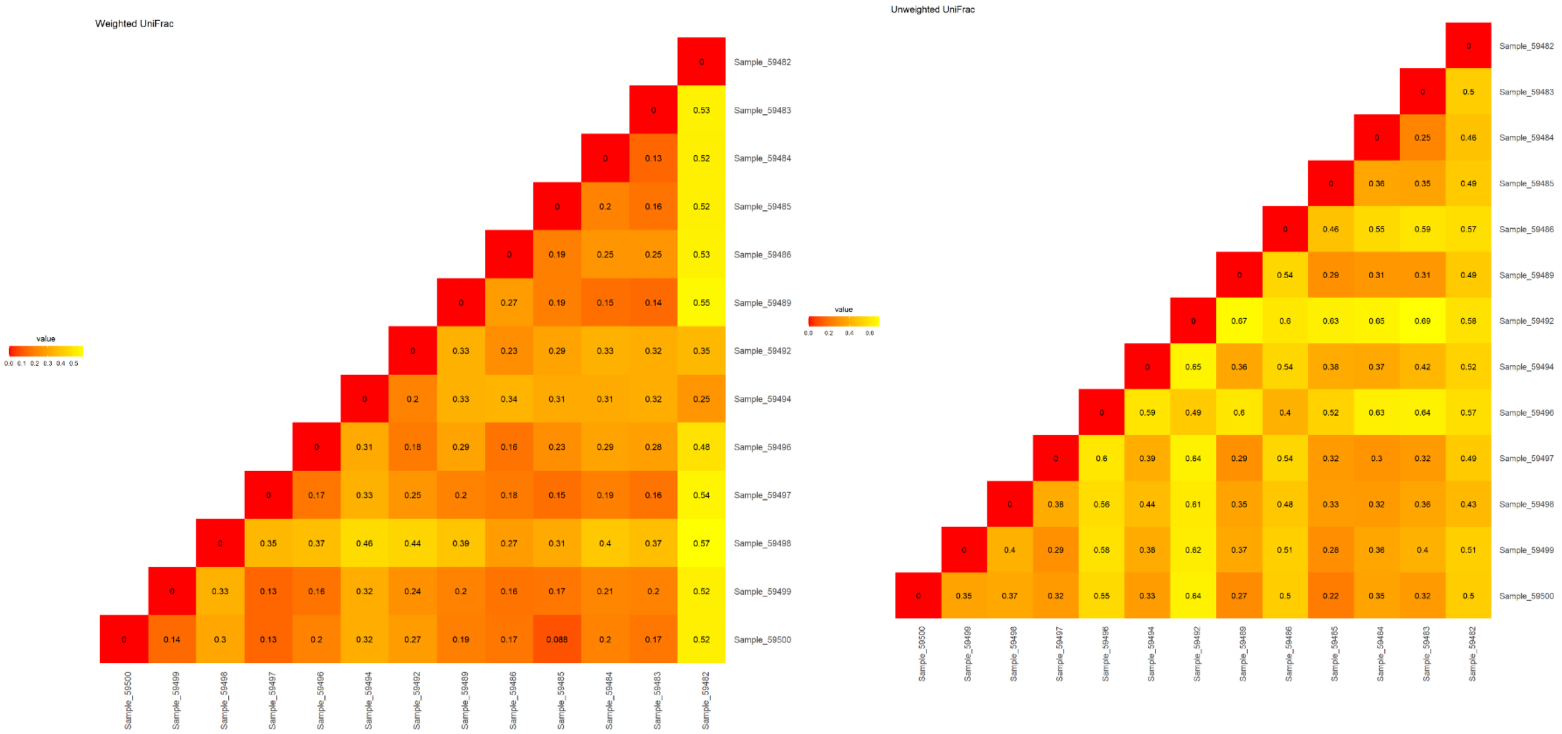

Figure S1. Heatmap of weighted UniFrac metrics (left) and unweighted UniFrac metrics (right) where the values represented in different color.

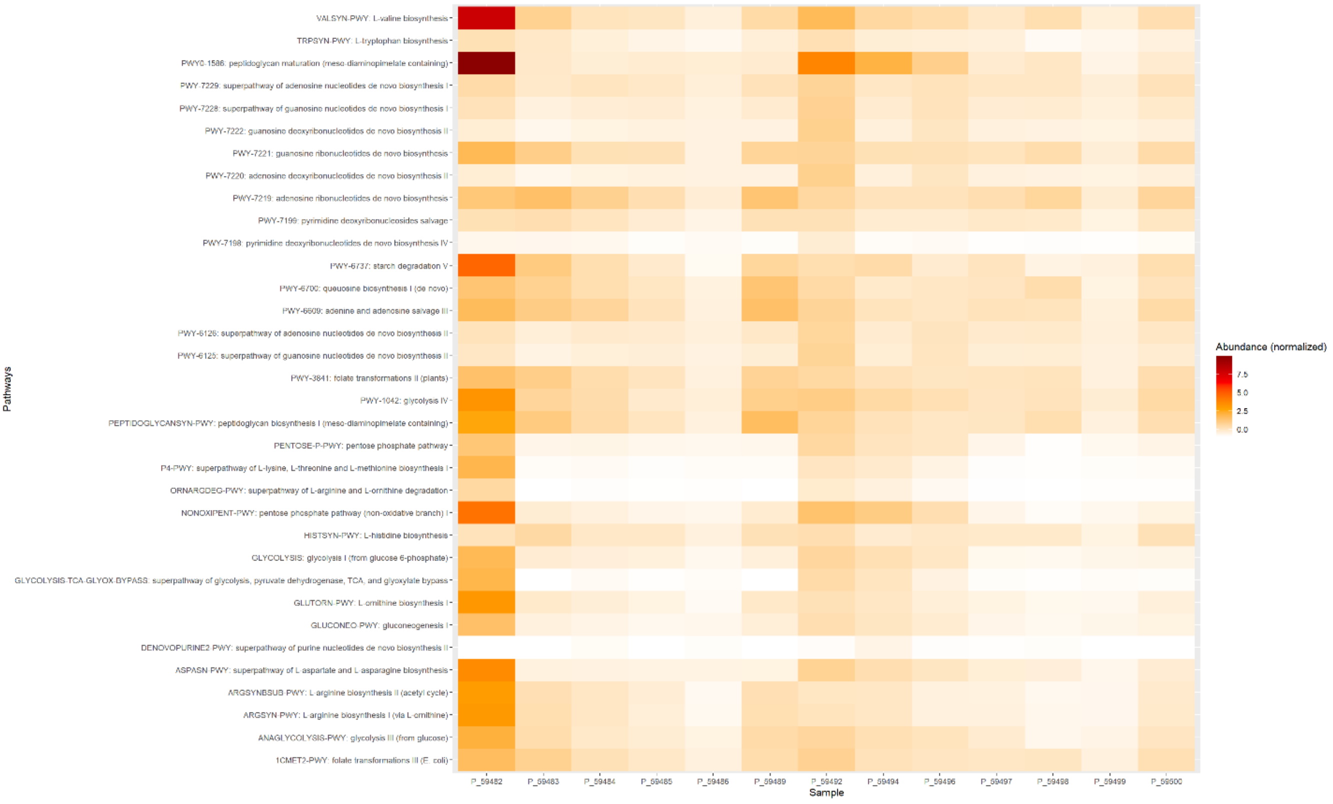

Figure S2. The functional characteristics of the gut microbiome in CRC patients' metabolic pathways where the different pathway names on the left and the abundance of each pathway in each patient are shown in color and are proportional to color intensity.

AIMS Press

© 2024 the Author(s), licensee AIMS Press. This is an open access article distributed under the terms of the Creative Commons Attribution License (http://creativecommons.org/licenses/by/4.0)
